# MicroRNAs’ control of cancer cell dormancy

**DOI:** 10.1186/s13008-019-0054-8

**Published:** 2019-10-10

**Authors:** Tatiana G. Ruksha

**Affiliations:** Department of Pathological Physiology, Krasnoyarsk State Medical University, P. Zeleznyaka str., 1, Krasnoyarsk, 660022 Russia

**Keywords:** Quiescence, Dormancy, Cancer, microRNA, Epigenetics

## Abstract

‘Dormancy’, in the context of carcinogenesis, is a biological phenomenon of decreased cancer cell proliferation and metabolism. In view of their ability to remain quiescent, cancer cells are able to avoid cell death induced by chemotherapeutic agents, and thereby give rise to tumor relapse at a later stage. Being a dynamic event, the dormant state is controlled by several epigenetic mechanisms, including the action of microRNAs. The present review highlights microRNAs that have been shown to be dysregulated in dormant cancer cells among different tumor types. MicroRNAs accomplish their control of cancer cell quiescence by targeting cell cycle regulators and signaling pathways involved in cell growth maintenance, including the AKT/phosphoinositide 3-kinase (PI3K) pathway. MicroRNAs, as components of intercellular vesicles, enable interactions to occur between cancer cells and cells of the microenvironment, resulting in the cancer cells either acquiring the quiescent state or, oppositely, stimulating them to proliferate. Taken together, the evidence obtained to date has collectively confirmed the involvement of microRNAsin cancer cell dormancy. Modulation of the various processes may enable optimization of the treatment of metastatic tumors.

## Background

In terms of carcinogenesis, dormancy may be identified as a biological state of tumors associated with a low proliferation rate and low metabolic activity. It is understood that a limited number of cancer cells reside in a quiescent state for an unspecified period until unknown stimuli induce their division, in addition to other biological activities that are specific to cancer cells. Epigenetic mechanisms are considered to fulfil the role of such stimuli. Among the various epigenetic mechanisms, the functioning of microRNAs results in translational repression or decay of target mRNAs. Each microRNA may have up to several hundred mRNA targets, and several genes may be regulated by one unique microRNA. The biogenesis, functioning and regulation of microRNAs have been thoroughly analyzed in numerous reviews [1–3]; therefore, the present review will not focus in any great detail on these aspects. Recently, it was shown that epigenetic therapies may affect dormant cells. The DNA-methylating agent, 5-aza-2′-deoxycytidine (5-aza-C), in combination with retinoic acid, was shown to restore orphan nuclear receptor expression, which regulates the gene expression of quiescent cells [4]. The hydroxymethylation enzyme, Tet2, was revealed to stimulate expression of the cell-cycle inhibitors,cyclin-dependent kinase inhibitor 1A (CDKN1A) andCDKN1B, resulting in dormancy of the melanoma cells [5]. Less is understood regarding the abilities of microRNAs to influence cancer quiescent cells. More importantly, it is not yet clear whether the dormant state of tumor cells should be shifted or not for therapeutic purposes. For that reason, the goal of the present review is to summarize the research data that have been published concerning the role of microRNAs in cancer dormancy, and their impact as modulators of tumor relapse and progression.

## Main text

### Tumor dormancy definition and methods used for quiescent cells identification

The first data concerning the role of microRNAs in cancer was published in 2002, when the research group of C. Croce reported on microRNA-15 (miR-15) and miR-16 downregulation in chronic lymphocytic leukemia cells [6]. Later it was discovered that mic are implicated in fundamental carcinogenesis-associated processes, including regulation via microRNAs targeting genes associated with the cell cycle, apoptosis, DNA repair, oncosupression, and cancer cell migration, invasion and evasion of the immune response.

Tumor cell quiescence may be defined as a reversible state of growth arrest in which cells enter the G_0_-phase of the cell cycle [7]. Dormant tumors characterized by a balance of apoptosis and cell proliferation results in the absence of progression and clinical symptoms. Cancer stem cells, which are determined as a cancer cell subpopulation responsible for tumor initiation and progression, can be dormancy competent, although not only cancer stem cells can become quiescent. Acquisition quiescence by cancer cells is a flexible process that can be induced by stress factors, including chemotherapy [8].

Core aspects of any study on cancer cell dormancy will include the choice of approaches that are used to characterize the dormant, quiescent state of the cancer cells, and to differentiate that pool of cells within the total population of cells in the tumor. Even though the phenomenon of tumor dormancy was first described in 1934 [9], a lack of unbiased methods for objectively studying this topic has restricted the quantity and the quality of the research results that have been obtained.

Cell cycle phase distribution monitoring via several standard procedures, including flow cytometric analysis to determine cells residing in the quiescent(G_0_) and gap 1 (G_1_) phases, may be used to investigate the population of dormant cells. Furthermore, cells that are resistant to various chemotherapeutic agents, and those which bear cancer stem cell markers, are currently being investigated. To confirm the proliferative state of cells, cell proliferation**-**specific markers, such as the Ki**-**67 antigen, have been used, as well as the incorporation of dyes [10]. As cellular quiescence is regulated by cyclins and cyclin**-**dependent kinases [11], this phenomenon may be investigated by estimating their protein and mRNA levels. Dormant tumor formation in vivo may be studied via the formation of small tumors that are stable in size, which develop within a lag period of time following injection of cancer cells into a model animal. Patient**-**derived xenograft tissues implanted into immunocompromised mice is one method that may be utilized for this process [12].

### MicroRNA profiling of quiescent cells

Measuring the expression of microRNAs is a standard approach for identifying the exact molecules that are crucial in the regulation of a process that is being investigated. MicroRNA profiling is used to characterize the unique expression pattern of quiescent cells. Almog et al. [13] performed an array scanning analysis in dormant tumor models of osteosarcoma and glioblastoma, where miR**-**190 was identified to be one of the most upregulated microRNAs. miR**-**190, overexpressed in cancer cells, formed microscopically sized tumors at the site of injection, which remained stable for up to 125 and 88 days for the osteosarcoma and glioblastoma tumor models, respectively. miR**-**190**-**overexpressed dormant microtumors were shown to contain proliferative cells, and miR**-**190 gene target and signaling pathway analyses revealed that miR**-**190**-**associated dormancy was associated with antigenic pattern expression redistribution on cancer cells, followed by immune escape. To identify microRNAs involved in regulating the shift of breast cancer cells from the dormant to the metastatic state, Giancotti et al. [14] constructed cDNA libraries originating from metastatic breast cancer cells. Subsequently, cDNA libraries were transduced into breast cancer 4TO7 cells that resided in the lungs, but did not give rise to metastasis. Murine microRNAs shown previously to give rise to breast cancer metastasis in the lung were divided into sets of up to 97 microRNAs transfected into 4TO7 cells, followed by injection into mice. The tumors that formed in mice were examined further for their microRNA expression levels, and miR**-**340, miR**-**346, and a combination of miR**-**138 and miR**-**223 were identified in each animal where breast cancer lung metastases had developed, indicating that the latter could be implicated in the transformation of breast cancer cells from the quiescent to the metastatic state. A similar study performed by Mori et al. [15] on gastrointestinal cancer stem cells separated them into CD13^**−**^/CD90^+^ proliferating cancer stem cells and CD13^+^/CD90^**−**^ dormant cancer stem cells. The latter were characterized according to their downregulated expression of miR**-**101 compared with CD13^**−**^/CD90^+^ cells. Bioinformatic analysis revealed that miR**-**101 was implicated in the cytokine/chemokine regulatory pathway (specifically, interleukin**-**8 and CXCL5), which again demonstrated the involvement of the immune system in the mechanism of maintenance of dormancy of cancer stem cells. The CD13^+^/CD90^**−**^dormant cancer stem cells were further characterized by their association with regulation of the tumor protein p53 (TP53) signaling pathway, which is involved with chemoresistance development upon treatment with genotoxic agents.

Dormant cancer cells residing in osteosarcoma, glioblastoma, breast cancer and liposarcoma in vivo models were established by Abdollahi et al. [18]. All of these tumor types were characterized by high proliferation and apoptotic rates, as well as impaired angiogenesis. MicroRNA profiling revealed similar microRNA expression patterns in all of these tumor types, where 16 out of 19 microRNAs (84%) were upregulated compared with non**-**dormant tumors of the same origin. Restoration of the levels of the most upregulated microRNAs, miR**-**580, miR**-**588 and miR**-**190, in fast**-**growing glioblastoma cells resulted in tumor growth delay. Gene targets of the aforementioned microRNAs included tissue inhibitor of metalloproteinases 3, hypoxia**-**induced factor-1α, basic fibroblast growth factors and the K**-**Ras tumor oncogene, which are well known carcinogenesis-related genes.

### MicroRNAs and their target genes are implicated in proliferation-quiescent state maintenance

To further elucidate the role of microRNAs in cancer cells’ shift from dormancy to the proliferative state, Watson et al. [17] restored expression of the miR**-**200a/200b/429 cluster in the murine mammary tumor cell line. The miR**-**200a/200b/429 cluster, which is involved in regulating the proliferation and migration of stem cells by targeting mesenchymal transcription factors, belongs to a microRNA family that is highly conserved among vertebrates. Reconstruction of the expression of the aforementioned microRNAs in the murine mammary tumor cell line resulted in a decrease in cell proliferation, as well as conversion of the cells’ morphology into the epithelial type. Furthermore, it led to a diminution of the levels of the transcription factors Snail, Twist1, Twist2, and Zeb1, and induced tumor transition to the state of dormancy, as determined by the formation of small size tumors in vivo. miR**-**200c has previously been shown to be involved in maintenance of the dormant state of osteosarcoma tumors. In the study by Tiram et al. [18], the osteosarcoma cell line was generated by using non**-**tumorigenic osteosarcoma cells, which gave rise to small tumors that did not exhibit any signs of angiogenesis and were stable in terms of tumor volume for a time period lasting up to 140 days in mice. miR**-**200c was one of the microRNAs that were differently expressed in dormant**-**tumor**-**forming cells compared with fast**-**growing ones, and its overexpression was associated with a prolonged survival rate in vivo. Therefore, these aforementioned data demonstrates miR-200 family involvement in tumor dormancy regulation.

Several experiments have been aimed at elucidating the mechanisms underlying the transition of cancer cells between the proliferative and dormant states. ΔNp63α, which is an isoform of p63 protein, was shown to be implicated in mammary stem cell quiescence. In MCF7 luminal estrogen**-**receptor**-**positive breast cancer cells, ΔNp63α stimulated reversible growth arrest, indicating its role in dormancy state development. MiR**-**205 was shown to be the most upregulated microRNA in MCF7 cells overexpressing ΔNp63α. The application of anmiR**-**20**-**specific mimic led to a decrease in the cell proliferation rate, and caused dormancy**-**associated protein dysregulation, including components of the bone morphogenetic protein signaling pathway target genes, inhibitor of DNA binding 1 (ID1) and ID3,andan inhibitor of this pathway, bone morphogenetic protein and activin membrane bound inhibitor (BAMBI) [19].

Cancer cell quiescence is also maintained by heat**-**shock proteins, of which heat**-**shock protein90 (Hsp90) has been shown to regulate protein function associated with cancer cell ‘stemness’ and quiescence. In that regard, Hsp90**-**specific inhibitors were revealed to induce a decrease in the expression of the cancer stem cell markers, CD44, and aldehyde dehydrogenase (ALDH). In addition, microRNA expression profiles were subjected to alterations when microRNAs involved in the regulation of signaling pathways such as those connected with mitogen**-**activated protein kinase (MAPK), β**-**catenin, and cell cycle/proliferation were revealed to be dysregulated [20].

Several studies have shown that miR-221 and miR-222 act as dormancy regulators. Acute lymphoblastic leukemia cells co**-**cultured with bone marrow stromal cells and primary human osteoblasts demonstrated decreased expression levels of miR**-**221 and miR**-**222, microRNAs that were described previously as leukemia cell proliferation regulators targeting the cell cycle inhibitor, CDKN1B (or p27). Previously, it was shown that p27 is involved in the quiescence of acute lymphoblastic leukemia cells [21]. Targeted downregulation of miR**-**221 and miR**-**222, followed by p27 overexpression, resulted in cell cycle arrest and an increase in chemotherapy resistance in the presence of bone marrow niche components, including stromal cells and osteoblasts, as well as independently of these cells. Modulation of miR**-**221 and miR**-**222 was also revealed to trigger quiescence of leukemia cells [22]. Similar results were obtained in glioblastoma cells, where the levels of miR**-**221 and miR**-**222 were shown to be increased in cells as they were entering into the cell cycle. Again, p27, and also p57, were validated as direct gene targets of these aforementioned microRNAs. Transfection with antimiR**-**222 inhibitor resulted in an increased number of cells residing in G_0_ phase. By contrast, miR**-**221 and miR**-**222 were shown to stimulate cells to enter the synthesis (S) phase, followed by apoptotic death. Anti**-**miR**-**222/223 transfection in mesenchymal stem cells transformed them to dormant cancer cells via exosomes and gap junctions, which resulted in a moderation of the proliferation rate of cancer cells and prolonged survival rates in vivo [23]. Therefore, low levels of miR-221 and miR-222 in quiescent cancer cells are considered to act as a mechanism that protects against an undesired shift from the quiescent to the proliferative state, prior to the onset of apoptosis [11].

Turning our attention to bone marrow breast cancer cells, these are characterized by the CD44^+^/CD24^**−**^ expression pattern. MiR**-**23b originating from bone marrow increased the number of CD44^**−**^ cells, thereby showing its potential to induce breast cancer dormancy, as CD44^**−**^ cells were revealed to have a decreased proliferation rate, reduced invasive capabilities, as well as being less sensitive to the chemotherapeutic agent, docetaxel [24]. MiR**-**424 and miR**-**503 were able to affect the cell cycle, leading to an increase in the number of G_1_ cells in colon cancer and osteosarcoma cells by inhibiting Cdc25A protein, which has an important role in cell cycle**-**dependent kinase activation [25]. Taking together, these reports indicate the impact of microRNAs on cell cycle-related genes, resulting in entry to the cell cycle or its arrest.

### MicroRNAs support interactions between proliferating/quiescent cells and microenvironment

**Fig. 1:**
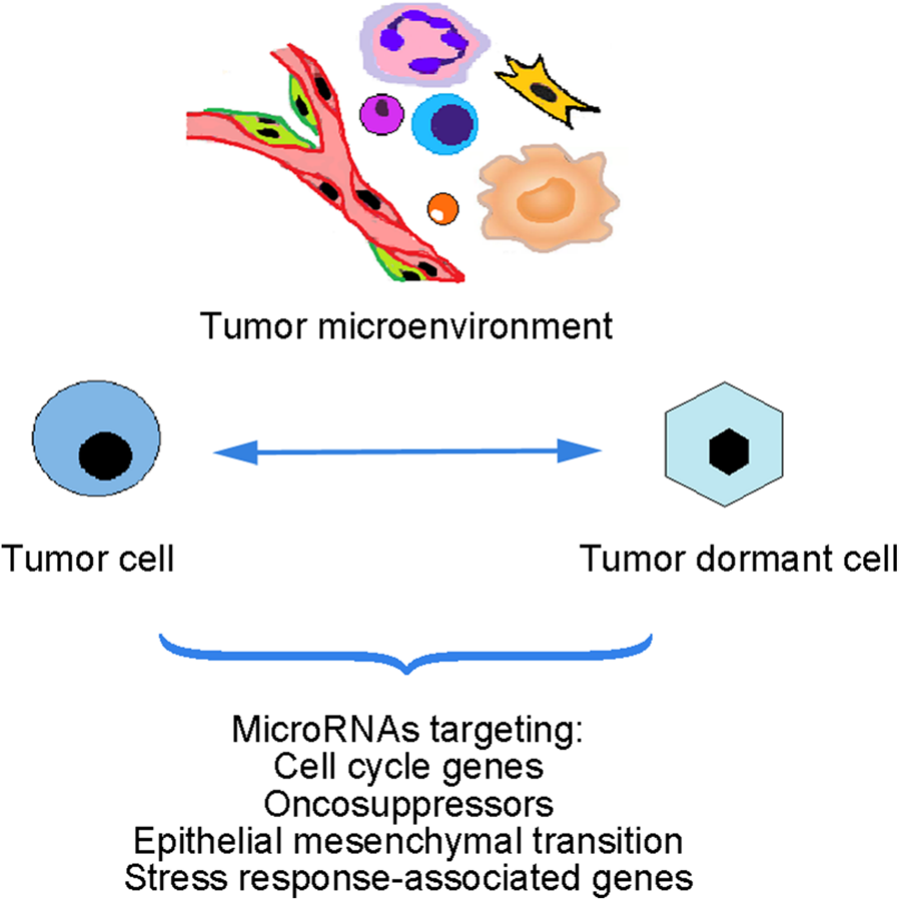
Summary diagram of microRNAs implicated in tumor cell quiescence

The tumor microenvironment itself is considered to be actively involved in carcinogenesis. Its components communicate via microRNAs with cancer cells, resulting in their switch from a dormant to a proliferative state (Fig. 1). Wells et al. [26] demonstrated that the microRNA profile of breast cancer cell**-**derived exosomes differed considerably from that of hepatic niche**-**derived exosomes. MicroRNAs derived from the latter were able to modulate cell proliferation of breast cancer cells, triggering several signaling pathways, and restoring the epithelial**-**type morphology of cells via induction of the re**-**expression of the epithelial marker, E**-**cadherin [26]. Bone marrow is an important site for quiescent breast cancer cells. Mesenchymal stem cells communicate to distinct breast cancer cells subsets differently, demonstrating bimodal effects on populations of regulatory T cells (Tregs) and T helper 17 (Th17) cells, and on interleukin**-**17 levels [27]. Mesenchymal stem cells and breast cancer cells communicate via gap junctions and exosomes, both of which are characterized by a specific microRNA profile. MiR**-**222/223, as mentioned above, are involved in cancer cell drug resistance [28], and these microRNAs were revealed to be overexpressed in mesenchymal stem cell**-**derived exosomes, which indicates their ability to act as signal transducers for a dynamic state of cancer cells upon microenvironmental stimuli.

MiR**-**126 is overexpressed in normal stem cells and progenitor hematopoietic cells, and is associated with an increased number of dormant leukemia stem cells. As endothelial cells were shown to express the highest rates of miR**-**126 among the components of the tumor microenvironment, communication between these cells and leukemia stem cells was inferred. Co**-**culture of endothelial cells and leukemia stem cells confirmed the occurrence of miR**-**126 trafficking from endothelial cells to leukemia stem cells via extracellular vesicles. Modulation of the expression level of miR**-**126 in leukemia cells resulted in an increase in their proliferation and apoptosis rates, as well as diminished colony formation. MiR**-**126 downregulation in endothelial cells and chronic myelogenous leukemia stem cells induced antitumor effects of tyrosine kinase inhibitors, a phenomenon that was determined via their increased survival rates in *miR126a***-**knockout mice [29]. As has been demonstrated in hematopoietic stem cells, miR**-**126 may regulate the expression of several components of the phosphoinositide 3**-**kinase (PI3K)/AKT signaling pathway [30], which was defined as an essential component of dormant estrogen**-**dependent breast cancer cells [31].

### Impact of microRNA level alterations on cell quiescence

There is no clear evidence to suggest if the shift between the quiescent and proliferative states can be considered a therapeutic goal. Therefore, gain-and-loss of function studies may help to provide further understanding. Pancreatic ductal adenocarcinoma stem cells resistant to chemotherapeutic agents were revealed to comprise the population of quiescent cells that are characterized by resistance to gemcitabine, predominantly existing in the G_0_ and G_1_, but not the S, cell cycle phases, increased levels of CDKN1A and CDKN1C, and decreased levels of cyclin D1. MicroRNA profiling revealed the miR**-**17**-**92 cluster to be downregulated in gemcitabine**-**resistant cells compared with vehicle**-**treated pancreatic ductal adenocarcinoma stem cells, whereas the levels of the miR**-**17**-**92 cluster were upregulated in pancreatic cancer bulk tumor tissues. MiR**-**17**-**92 levels were decreased in differentiated pancreatic cancer cells, leading to an enhancement in the level of the stem cell marker, CD133, as well as an increase in the number of cells in G_0_/G_1_ phase. Furthermore, anmiR**-**17**-**92 inhibitor stimulated tumor growth in vivo. Subsequent gene target analysis revealed that the miR**-**17**-**92 cluster activated the cell**-**cycle regulator p21, activin receptor**-**like kinase 1 (ALK1), and the transcription factor, TBX3, which is implicated in cell cycle and cancer progression control.TBX3 itself influenced the transforming growth factor**-**β1 signal pathway [12]. In summary, the identification of microRNAs, which can be key regulators of cancer quiescence, could be useful to prevent minimal residual disease [8].

## Conclusions

To conclude, several mechanisms underpinning the involvement of microRNAs in regulation of the quiescent state of cancer cells, and their shift to a proliferative state, have been elucidated (Table 1). MicroRNAs take part in the quiescent-proliferative state of cancer cells and its dynamic balance. Proper microRNA profiling for various cancer types is required to determine if specific microRNAs of quiescent cancer cells exist. Further functional studies may help to elucidate specific roles of these epigenetic regulators in tumor dormancy. Therapeutic strategies targeting microRNAs may be hypothesized, but their usefulness and applicability require validation by further research, including in vivo studies, before any firm conclusions may be reached. A broadening of the experimental systems investigated to include other cancer types, both in vitro and in vivo, is required, as the majority of the studies performed to date have been conducted on breast cancer and leukemia models. An improved understanding of the diversity of cancer cells, and their associated sensitivities to chemotherapeutic agents, will optimize personalized therapy approaches, which are necessary if cancer survival rates are to be improved.

**Table 1:** Characteristics of cancer cell quiescent state-associated microRNAs

MicroRNA	Cancer type involved/definition used to describe cells studied	Method used for quiescent cell identification	Target/key biological events regulated	References
miR-17-92	Pancreatic cancer/quiescent cancer stem cells	Cell cycle analysis by flow cytometry, Ki-67 negative cells identification by immunocytochemistry	p21, ALK1, and transcription factor TBX3/G_0_-to-G_1_ phase transition enhancement	[14]
miR-101	Liver cancer/liver cancer stem cell CD13^+^/CD90^−^ subpopulation	Fluorescence activated cell sorting	JARID1A, JMJD1B, TP53INP1, EZH2/stem cell pluripotency	[15]
miR-126	Chronic myelogenous leukemia/chronic myelogenous leukemia stem cells	Fluorescence activated cell sorting	PI3K/AKT signaling pathway components/proliferation, apoptosis, and colony formation	[29]
miR-190	Osteosarcoma, glioblastoma/dormant cells	In vitro proliferation assay based on cellular DNA content measurement, Kaplan–Meier analysis and Ki-67 immunohistochemistry for dormancy models in vivo	Nuclear factor I/B, human T cell leukemia virus type I binding protein 3/cell proliferation	[13]
miR-200a/200b/429 cluster	Breast cancer/low proliferative cells	Cell proliferation measured by immunofluorescent detection of anti-histone H3 antibodies	Snail, Twist1, Twist2, Zeb1/cell proliferation	[17]
miR-200c	Osteosarcoma/dormant-tumor-forming cells	Kaplan–Meier analysis for survival	Tissue inhibitor of metalloproteinases 3, hypoxia-induced factor 1α, basic fibroblast growth factor, K-Ras/metastasis	[18]
miR-205	Breast cancer/G_0_ quiescent cancer stem cells	Flow cytometry for cell cycle analysis, 5‐bromo‐2′‐deoxyuridine-Ki-67 double immunostaining	Bone morphogenetic protein signaling pathway target genes (ID1 andID3), and the inhibitor of this pathway, BAMBI/induction of quiescence in cells	[19]
miR-221	Acute lymphoblastic leukemia/quiescent cells	Cell proliferation evaluation by flow cytometry, p27 immunostaining	The cell cycle inhibitor, cyclin-dependent kinase inhibitor 1B (or p27)/cell cycle regulation, sensitization to cytotoxic agents	[22]
miR-222	Acute lymphoblastic leukemia, breast cancer/quiescent cells	Flow cytometry for cell cycle analysis, 5-bromo-2′-deoxyuridine staining	Cell cycle inhibitor cyclin-dependent kinase inhibitor 1B, p27/drug resistance	[21, 14]
miR-223	Breast cancer/breast cancer dormant stem cells	Flow cytometry for cell cycle analysis, Ki-67 immunostaining	Drug resistance enhancement to carboplatin	[14]
miR-424	Colon cancer, osteosarcoma/proliferating cancer cells	Flow cytometry for cell cycle analysis	Increase in the number of G_1_ cells	[25]
miR-503	Colon cancer, osteosarcoma/proliferating cancer cells	Flow cytometry for cell cycle analysis	Cyclin D, cyclin E, Cdc25A/Increase in the number of G_1_ cells, cell cycle arrest via targeting M-phase inducer phosphotase	[25]
miR-580	Osteosarcoma, glioblastoma, breast cancer, liposarcoma/quiescent cells	Tumor volume analysis, proliferation assay based on cellular DNA content measurement, Ki-67 immunostaining	Tissue inhibitor of metalloproteinases 3, hypoxia-induced factor-1α, basic fibroblast growth factor, K-Ras/induction of antiangiogenic and dormancy promoting pathways EphA5, Angiomotin	[16]
miR-588	Osteosarcoma, glioblastoma, breast cancer, liposarcoma/quiescent cells	Tumor volume analysis, proliferation assay based on cellular DNA content measurement, Ki-67 immunostaining	Tissue inhibitor of metalloproteinases 3, hypoxia-induced factor-1α, basic fibroblast growth factor, K-Ras/induction of antiangiogenic and dormancy promoting pathways EphA5, Angiomotin	[16]

*AKL1* Activin receptor-like kinase 1, *PI3K* phosphoinositide 3-kinase, *ID1/3* isopentenyl-diphosphate Δ-isomerase 1/3, *BAMBI* bone morphogenetic protein and activin membrane bound inhibitor

## Data Availability

Not applicable.

## Abbreviations

ALDH: aldehyde dehydrogenase
MAPK: mitogen-activated protein kinase
Th: T helper cell
Treg: regulatory T cell
